# Clinical and epidemiological characteristics of SARS-CoV-2 virus in ambulatory children under 2 years old

**DOI:** 10.3389/fped.2022.957273

**Published:** 2022-11-29

**Authors:** Carolina A. Perez, Ivana Ormazabal, Javier Pérez-Valenzuela, Andrea Araya, Rafael A. Medina, Cecilia Perret

**Affiliations:** ^1^ Department of Pediatrics, School of Medicine, Pontificia Universidad Católica de Chile, Santiago, Chile; ^2^ School of Medicine, Pontificia Universidad Católica de Chile, Santiago, Chile; ^3^ Department of Pediatric Infectious Diseases and Immunology, School of Medicine, Pontificia Universidad Católica de Chile, Santiago, Chile; ^4^ Advanced Interdisciplinary Rehabilitation Register (AIRR) - COVID-19 Working Group, Faculty of Medicine, Pontificia Universidad Católica de Chile, Santiago, Chile; ^5^ Deparment of Microbiology, Icahn School of Medicine at Mount Sinai, New York, United States

**Keywords:** COVID-19, SARS-CoV-2, infant, toddler, pediatric, outpatient, ambulatory, characteristics

## Abstract

**Background:**

SARS-CoV-2 is an emerging virus that has mainly affected adults; hence, most clinical information has been derived from that population. Most pediatric cases are mild and with nonspecific symptoms requiring outpatient management. Children are a major source of spread for most traditional respiratory viruses. Their role in SARS-CoV-2 transmission was thought to be relevant. Children under the age of two comprise a group that is more susceptible to infection since vaccines have not been approved for them until recently. The knowledge of clinical manifestation of COVID-19 in young children is scarce.

**Objectives:**

To describe the clinical, epidemiological, and demographic characteristics of children under 2 years old with confirmed COVID-19, who did not require hospitalization.

**Methods:**

This descriptive study was performed from May, 2020 to June, 2021. Children ages 0–2 years with COVID-19, confirmed by transcriptase-polymerase chain reaction assay that were performed in laboratories of the Red de Salud UC CHRISTUS Health Network, were selected to be contacted. If the parents accepted participating and their children were not hospitalized, a survey was sent to the patients' caregivers.

**Results:**

Of the 242 cases, 159 caregivers answered the survey (65.7%). The median age of the subjects was 14 months, and 53.5% were males. Fifty percent had comorbidities, of which one third corresponded to atopy. Ninety eight percent were secondary cases. Most of them were infected within their households (81%). The most frequent sources were their parents, followed by their grandparents. The most common symptom was fever (78%) followed by irritability (67.3%), rhinorrhea (66%), and fatigue (64.8%). Infants less than 6 months old more often presented with conjunctival congestion and less loss of appetite compared to older children (*p* < 0.05).

**Conclusions:**

This study provides valuable insights regarding COVID-19 in ambulatory young children. Most cases of SARS-CoV-2 infection in children under 2 years old do not require hospitalization. There was a slight male predominance, and the majority had been infected within their households. SARS-CoV-2 infection should be suspected in children under 2 years old presenting with fever, irritability, fatigue, and rhinorrhea. Children with positive household contacts and fever should also be tested for COVID-19.

## Introduction

On December 31, 2019, the first case of severe acute respiratory syndrome coronavirus-2 (SARS-CoV-2) was identified in Wuhan, China (1). It is characterized by its high rate of contagion and potential to affect all age groups, including the pediatric population (2). In early March, 2020, the first case was identified in Chile, and on March 11th the World Health Organization (WHO) declared a SARS-CoV-2 pandemic (3).

In Chile, from March, 2020 to June, 2021, 1,858,307 confirmed and probable cases were reported, with a prevalence rate of 9,550.2 per 100,000 inhabitants. In pediatrics, 253,613 cases between the ages of 0–19 years were registered. In children under 4 years of age, 42,201 cases were reported, with a prevalence rate of 3,545.2 per 100,000 inhabitants. The age group with the most reported infections was adults between 25 and 35 years, and 8.33% corresponded to children under 15 years (4). This period included the first and third waves of the pandemic, one in May-June, 2020, and the other in April-June, 2021 (4). In the first wave, the B.1 Wuhan-like variant was the predominant virus circulating in Chile (5), while in the third wave it was the P1 (Gamma) variant of concern (6).

SARS-CoV-2 is an emerging virus that has mainly affected adults, of whom some can become severely ill, require hospitalization, and can even die from coronavirus disease 19 (COVID-19). Hence, most of the clinical information and guidelines are derived from this population (7), leaving its most frequent form of presentation in the pediatric population yet to be fully elucidated (2). Infections with SARS-CoV-2 in children have been described with higher proportion of asymptomatic cases, a faster recovery, and a better prognosis compared to infections in adults (8) and are usually characterized by milder and nonspecific symptoms (9). Additionally, most pediatric cases require only outpatient management (10).

Although this virus affects children less frequently, serious, and even fatal cases have also been described (2, 10). Some studies have reported up to 4% of severe cases (10), with higher mortality rates in children between 10 and 18 years old (11) and in infants and toddlers under 2 years old, up to 34% need hospitalization (12). Risk factors for developing severe illness among children infected with SARS-CoV-2 include age, chronic comorbidities, and high viral load (13).

There is a wide spectrum of clinical manifestations, from asymptomatic to severe presentations, such as multisystem inflammatory syndrome, associated with COVID-19. The range is even wider with the emergence of new variants that are in circulation, and the implementation of vaccination (14). Several studies suggest that children are an important source of infection for many traditional respiratory viruses, and that they play an important role in disease outbreaks and epidemics (3). Therefore, a similar transmission dynamic of SARS-CoV-2 in children was likely possible. Nevertheless, several studies on SARS-CoV-2 have revealed that the majority of these infections in children occur within the household (10). Infants and toddlers are currently more susceptible to infection and spreading the virus since, thus far, vaccines in some countries have only been approved for children over 3 years old (15).

The lack of information of the relationship between clinical patterns of acute respiratory infection and viral etiology could lead to fail in recognizing a case, and therefore a delay in proper treatment. In this context, it is important to generate improved guidelines of the clinical characteristics, including mild conditions, for the pediatric population affected by SARS-CoV-2. This would allow early detection of the disease, and prevent wide virus transmission; especially in children less than 2 years old who have not been vaccinated, and thus have increased vulnerability for contracting the virus from family members in their familial clusters (16).

The aim of this study was to describe the clinical, epidemiological, and demographic characteristics of children under 2 years old with confirmed COVID-19 who did not require hospitalization.

## Materials and methods

This was a descriptive study performed by pediatricians and pediatric infectious diseases specialists. Children between 0 and 24 months of age who were diagnosed with COVID-19 at the emergency rooms and outpatient centers of the Red de Salud UC CHRISTUS University Health Network, were selected to be contacted for participation in this study. Cases of COVID-19 were confirmed by nasopharyngeal and oropharyngeal transcriptase-polymerase chain reaction assay (RT-PCR) in the network laboratories. If the patients were hospitalized at the time of diagnosis or during the course of the disease, they were excluded from the study. If parents agreed to participate, a survey was sent by email to the patient caregivers, asking about epidemiological, demographic, and clinical manifestations. Once parents received the survey, they signed the Informed Consent form electronically. The recruitment was performed between May, 2020 and June, 2021.

The survey questionnaire was created by the investigators and reviewed by 9 specialists, from the area of Ambulatory Pediatrics, Infectious Diseases and Respiratory Pediatric Diseases of the Pontificia Universidad Catolica de Chile, who suggested changes regarding appropriate medical terminology and the type of questions to be included. Preliminary interviews were conducted with 10 caregivers who were not involved in the study to assess the internal validity of the questionnaire, and to make sure the survey was fully understood. Subsequently, corrections were made, and finally this last version was used in the study (See Annex S1 in Supplementary material). After this validation process, the questionnaire was sent to be filled out by the parents of the participants. The data collected included age, sex, comorbidities, and clinical manifestations of the disease for each subject.

The information obtained was registered in a database that was kept confidential, where identifiable information of the patient was anonymized through a computer application. This project was approved by the Scientific and Ethical Committee of the Pontificia Universidad Catolica de Chile (ID number 200119001).

The data was analyzed with the Statistical Package for the Social Sciences (SPSS) 26.0 software. Numerical variables were presented as median and range, and categorical variables as frequency and percentage. Considering that the data did not have a normal distribution, we performed non-parametric tests. For statistical analysis, we used the *U* Mann-Whitney test, *χ*^2^ test, and Fisher’s Exact test. A *p*-value < 0.05 was considered to be statistically significant.

## Results

### Demographic and epidemiological characteristics

Our study was conducted between May, 2020 and June, 2021, which was one of the periods of highest circulation of the SARS-CoV-2 virus in Chile, including the first and third waves of outbreaks, one in May-June, 2020, and the other in April-June, 2021. Eighty-one participants were enrolled in the study during the first wave, when the B.1 Wuhan-like variant was the predominant virus circulating in Chile. Twenty-eight participants were enrolled in the third peak's wave, in April-June 2021, when the dominant variant was Gamma. The positivity of the RT-PCR SARS-CoV-2 samples ranged from 0.4–26.6%, with a median of 4.4%. The highest positivity (26.6%) was reported in May, 2020.

**Figure 1:**
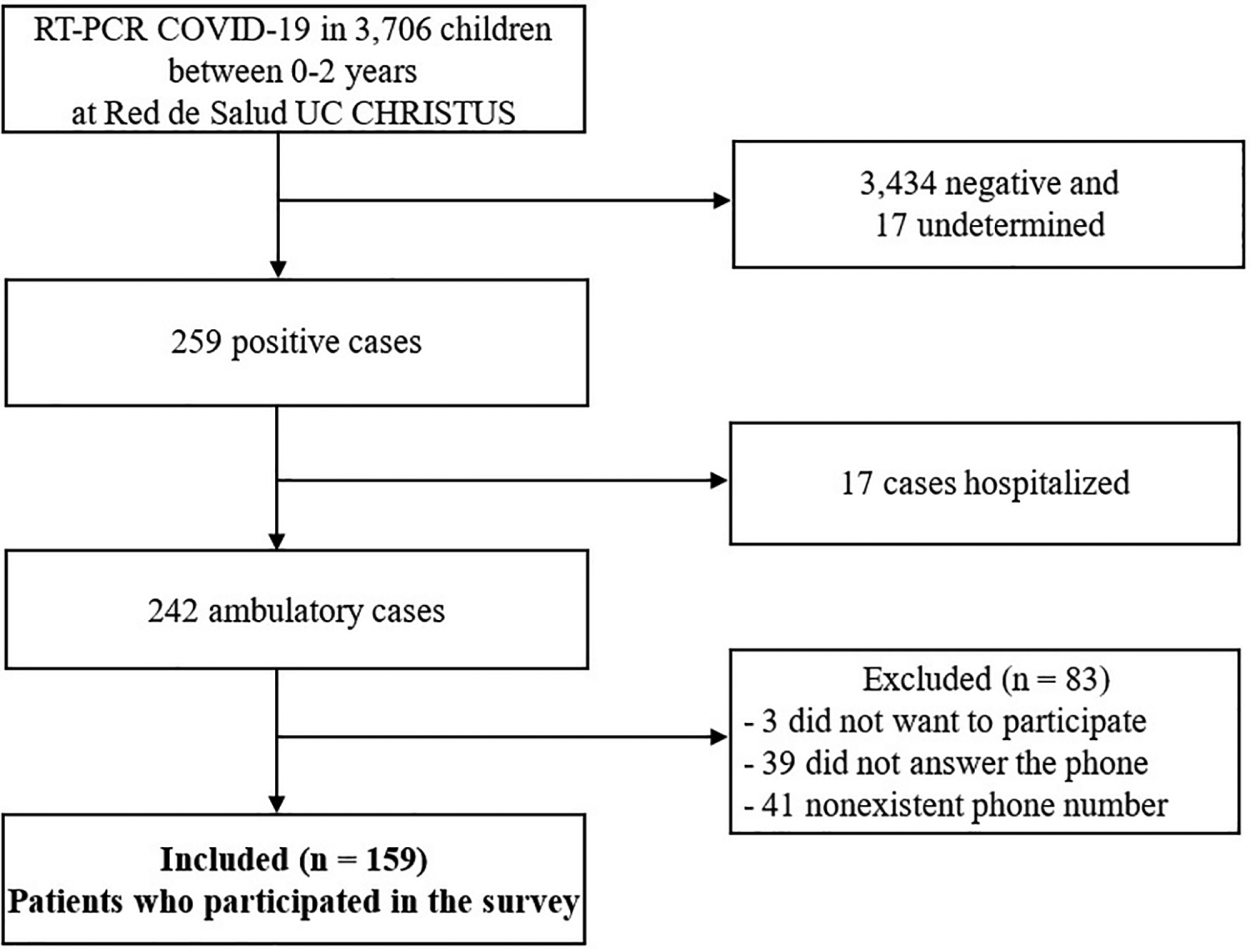
Flow chart of the study.

During this period, a total of 3,760 RT-PCR COVID-19 were performed in children between 0 and 2 years old in the Red de Salud UC CHRISTUS Health Network, of which 259 (7%) had positive results. Of these, 242 (94.3%) children were ambulatory, and 17 (5.7%) children were hospitalized, or had already been hospitalized for other reasons at the time of diagnosis. Of 242 participants, 201 were contacted, and 159 (65.7%) caregivers agreed to participate, and responded to the survey (Figure 1).

No statistically significant differences between the participants and the other outpatient children with confirmed COVID-19 were found with respect to age and sex. The participants were tested for COVID-19 in the emergency room in a greater proportion than the ambulatory children not included in the study, who were tested more often in ambulatory diagnostic centers.

Table 1 shows the general characteristics of the participants. The median age was 14 months (1–24 months), and 53.5% were males. The neonates were not included in this study, as were all hospitalized for follow-up when they were diagnosed with COVID-19. This was a recommendation by experts due to a lack of knowledge at that time, of how seriously ill this age group could become. Fifty percent of the children had comorbidities, 1/3 of which corresponded to atopy. Eighty eight percent of the participants had an immunization plan done corresponding to their age. Regarding vaccination against influenza, 60% were complete, 25% were incomplete (one out of 2 doses), 13% were not of age as they were under 6 months, and 2% of the caregivers did not answer the question.

**Table 1:** Demographic characteristics in 159 children, less than two years of age with COVID-19.

Characteristic	Total (*n* = 159)
**Age (months), median (range)**	14 (1–24)
**Sex (male), *n* (%)**	85 (53.5)
**N° of children in the family, median (range)**	2 (1–5)
**Presence of comorbidities, *n* (%)**	81 (50.9)
**Comorbidity categories, *n* (%)**	
– Atopy^a^	50 (31.4)
– Prematurity	22 (14)
– Malnutrition	23 (14)
– Others	11 (7)
**Previous hospitalizations, *n* (%)**	90 (38)
**Complete immunization status, *n* (%)**	140 (88)

^a^ Atopy: atopic dermatitis, allergic rhinitis, asthma.

All of the participants had documented history of exposure to a confirmed positive case, with 130 of 159 (81.7%) of the patients having at least two COVID-19 contacts. Most of the subjects were infected by a household member (81%; *n* = 129). The most frequent source was one or both parents (93%), followed by grandparents (33.3%) and siblings (29.5%). During the complete lockdown in Santiago, which occurred during the study period, only 16% of the parents were able to maintain confinement at home prior to the child's infection. On the other hand, 25.2% of the children were exposed outside of their homes, mainly by other relatives, neighbors, and/or family friends. Only 3 children were infected at a day care facility.

### Clinical features

**Figure 2:**
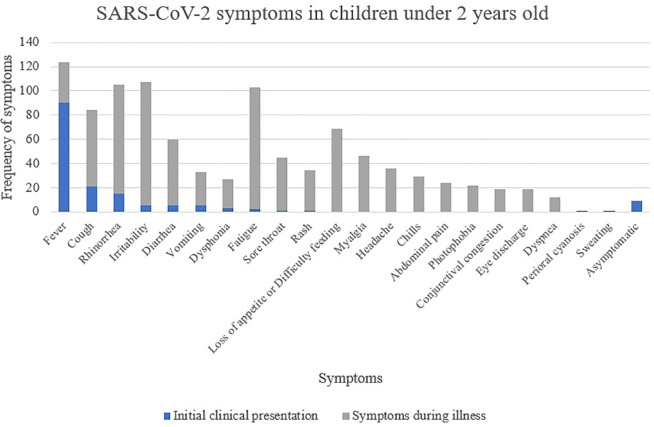
Clinical features at initial presentation and during illness in 159 children with COVID-19.

There was a wide spectrum of clinical manifestations, summarized in Table 2 and Figure 2. Fever was the most frequently reported symptom at initial presentation (*n* = 90, 56.6%) and during illness (*n* = 124, 78%). There were no other predominant initial symptoms besides fever. Of the children who had fever, thirty-five (28.2%) had temperature >39°C through the disease evolution. Four participants had only fever during the illness, of whom all were over 12 months of age.

**Table 2:** Clinical features at initial presentation in 159 pediatric patients with COVID-19.

Initial clinical presentation	*n* (%)
Fever	90 (56.6)
Cough	21 (13.2)
Rhinorrhea	15 (9.4)
Asymptomatic	9 (5.7)
Irritability	5 (3.1)
Vomiting	5 (3.1)
Diarrhea	5 (3.1)
Dysphonia	3 (1.9)
Fatigue	2 (1.3)
Sore throat	1 (0.6)
Perioral cyanosis	1 (0.6)
Rash	1 (0.6)
Sweating	1 (0.6)

Other common symptoms during the infection were irritability (67.3%), rhinorrhea (66%), and fatigue (64.8%). Cough appeared in only 50% of the total cases, and, of these, 67 children had irritative cough. Eighty-five children (53.5%) had gastrointestinal symptoms, including diarrhea (*n* = 60, 37.8%), vomiting (*n* = 33), and abdominal pain (*n* = 24). Respiratory distress was present in 7.5% of the cases. Nine children (5.9%) were asymptomatic at the time of the diagnosis, but two of them developed symptoms later on. Symptoms in the different age groups are shown in Table 3.

**Table 3:** Clinical features during illness, in 159 children less than 2 years of age with COVID-19, according to age group.

Symptoms during illness	0–6 months *n* (%)	>6–12 months *n* (%)	>12–24 months *n* (%)
Number of patients	32 (20.1)	39 (24.5)	88 (55.3)
Fever	22 (68.8)	33 (84.6)	69 (78.4)
Irritability	20 (62.5)	28 (71.8)	59 (67)
Fatigue	20 (62.5)	24 (61.5)	59 (67)
Rhinorrhea	19 (59.4)	27 (69.2)	59 (67)
Cough	15 (46.9)	21 (53.8)	48 (54.5)
Loss of appetite or Difficulty feeding	3 (9.4)	17 (43.6)	49 (55.7)
Diarrhea	10 (31.3)	14 (35.9)	36 (40.9)
Myalgia	13 (40.6)	11 (28.2)	22 (25)
Headache	8 (25)	9 (23.1)	19 (21.6)
Dysphonia	8 (25)	8 (20.5)	11 (12.5)
Sore throat or Dysphagia	7 (21.9)	14 (3.9)	24 (27.3)
Rash	4 (12.5)	9 (23.1)	21 (23.9)
Vomiting	5 (15.6)	11 (28.2)	17 (19.3)
Abdominal pain	6 (18.8)	6 (15.4)	12 (13.6)
Conjunctival congestion	8 (25)	5 (12.8)	6 (6.8)
Eye discharge	7 (21.9)	4 (10.3)	8 (9.1)
Photophobia	7 (21.9)	3 (7.7)	12 (13.6)
Dyspnea	2 (6.3)	3 (7.7)	7 (8)
Chills	2 (6.3)	12 (30.8)	15 (17)
Asymptomatic	3 (9.4)	0 (0)	4 (4.5)

In a subgroup analysis, we compared the presence of symptoms in infants aged 0–3 months, 3–6 months, 6–12 months, and children older than 12 months of age. Fever was the most common symptom in all subgroups (44.4%, 78.3%, 84.6% 78.4% respectively), with no significant differences between them. In the 0–3 and 3–6 months old groups, loss of appetite was less reported compared to in the 6–12 months group (*p* < 0.039) and children older than the 12 months group (*p* < 0.001). In the 3–6 months old group there was higher presence of conjunctival congestion compared to the children older than 12 months (*p* < 0.01). No statistically significant differences were reported for other symptoms.

Symptoms presented during the first and the third waves of outbreaks were compared. Fever was the predominant symptom in both waves, with no differences between them. Irritability, fatigue, feeding difficulty, odynophagia, and headache, were statistically more frequent, while myalgia and abdominal pain were less frequent during the third wave than the first one (See Annex S2 in Supplementary material).

Of the 159 patients surveyed, only in 9 children a respiratory molecular panel including other respiratory viruses was performed, of which one had a rhinovirus-enterovirus co-infection.

## Discussion

The purpose of this study was to describe the clinical, epidemiological, and demographic characteristics of a cohort of children between 0 and 2 years old with confirmed COVID-19 who did not require hospitalization. To our knowledge, there are no published epidemiological studies in Latin America regarding COVID-19 in pediatric outpatient care. Our results indicate that SARS-CoV-2 is a mild infection in children between 0 and 24 months of age, since most cases did not require hospitalization during the illness, as had been described previously (10). In our study population we found a slight male predominance, consistent with other reports (9, 17).

SARS-CoV-2 is a highly contagious virus, that is transmitted mainly from person to person (10). In our study, all the participants had documented history of exposure to a confirmed positive case. In agreement with previous studies, our results showed that the most frequent source of exposure was within the household, with parents being the most frequent primary case (18, 19). However, the second most common source of transmission was the grandparents, while in the other studies cited, it was the siblings. This difference could be due to the way Latin American families are conformed, with grandparents commonly part of the household. These findings should be interpreted with caution since exposure to other sources has been limited due to social distancing strategies and lockdown measures implemented in many countries during the pandemic (10). Our findings reinforce that infants and toddlers do not represent a major source of transmission, but instead, can become infected by their family group. As preventive measures, several countries had established prolonged maternity leave policies, online school classes, working from home, and closed day care centers during the first and third waves of the pandemic. Consequently, children stayed at home, thus facing a higher risk of becoming infected by household contacts (20). In recent months there has been a tendency to reduce or stop previous control strategies, which could lead to changes in the transmission patterns. Nevertheless, some studies demonstrate that the predominance of household exposure is maintained for children (18). Considering this, our data suggest that day care centers should reopen, or remain open with adequate preventive measures.

There is scarce information regarding the clinical presentation of SARS-CoV-2 infection in children who are under 2 years of age (17), and most of the studies include hospitalized adults (7). These studies highlight that fever remains the cardinal symptom at the onset and throughout the course of the illness. However, it is important to notice that it is usually a low-grade fever (17, 19). Interestingly, we found out that rhinorrhea was a common symptom in children, while in adults it is reported in less than 10% of patients (21). Other frequent manifestations in children between 0 and 2 years were irritability and fatigue, more often occurring than has been reported in previous studies (9, 22). On the other hand, cough was not an important symptom in this population, and was reported in only half of the patients during evolution of the disease. Additionally, unlike adults (21, 22), children presented with diarrhea more frequently, whereas dyspnea was reported less often. Noteworthy in our study, is that infants under 6 months of age had less loss of appetite or feeding difficulties than children between 6 months and 2 years, which could be explained by the fact that children under 6 months are likely to still be breastfed.

A major topic of ongoing research is how the emergence of the new SARS-CoV-2 variants impacts a patient's clinical presentation and outcome. New variants have been associated with increased transmission, different clinical features, more severe cases in some instances, and a reduced response to COVID-19 vaccines (14, 23). Fever has been shown to be the most commonly reported symptom, and other symptoms vary depending on the virus variant that predominates at specific point in time (19, 23). However, most of the pediatric cases have not required hospitalization during the different waves of the pandemic, which our study is in agreement (19, 23). Therefore, depending on the variant circulating at a given time, the clinical spectrum of manifestations might vary, as our results also indicate to be. Hence, we must be alert to early detection of new variants and their correlation with severity. Further studies considering contemporary SARS-CoV-2 variants and prevention strategies are needed in order to better understand COVID-19 symptoms in very young children.

Co-infection with other respiratory viruses could not be assessed in all the participants since only 9 of them underwent a broader etiological study. However, during the study period, the circulation of other respiratory viruses was not detected Chile, so it is very unlikely that coinfections were occurring at that time (24). Recent studies, after the emergence of the other respiratory viruses, have shown that respiratory syncytial virus co-infections can modulate COVID-19 severity, with the need for a higher level of care, longer length of hospital stay and progression to acute respiratory distress syndrome (25).

Half of the patients had a comorbidity, which was much higher than the 20% of comorbidities reported in other pediatric studies (17, 22). Thirty-three percent of the children had atopy, such as allergic rhinitis, asthma, and atopic dermatitis. Current data support a lower risk of acquiring SARS-CoV-2 infection in allergic children, although this clinical association has not been fully elucidated (26, 27). It should be noted that the number of participants with allergies reported in our study could have been overestimated given that some symptoms of allergy and COVID-19 infection overlap, resulting in more frequent testing of this group of children than of those without these comorbidities.

This study has some limitations. Firstly, our results correspond to data obtained from a single Health Center, which could represent a bias of the population studied. However, the Red de Salud UC CHRISTUS is one of the largest health networks in Chile, which includes laboratory centers located in various subdivisions of the city of Santiago, and in other cities of the country. Patients with COVID-19 confirmed by PCR were from 29 out of the 40 municipalities of Santiago, creating a representative sample in the country's capital. Future investigations could be designed to include additional cities, separating the analyses of urban and rural areas and comparing different socioeconomic levels. Secondly, there was not a validated questionnaire available for the purpose of our study. Therefore, we created an ad-hoc questionnaire that was reviewed by some pediatric specialists in the area of Ambulatory Pediatrics, Infectious and Respiratory Pediatric Diseases. Subsequently it was evaluated through preliminary interviews with ten caregivers to evaluate that the questionnaire was fully understood and to assess internal validity.

Another aspect to be considered is that the data was collected from the parents of the patients two weeks after their infections, which could have introduced a recall bias. Despite this, our results indicate that contacting the parents 14 days after the diagnosis allowed us to collect comprehensive information of all the symptoms, and the outcomes of COVID-19 in the participants. Finally, since the children enrolled in this study were attended in an emergency room (ER) more often than in an ambulatory laboratory center to be tested for SARS-CoV-2, compared to those who were not surveyed, this could be a bias in the selection of participants towards those whose symptoms were more alarming to their parents.

## Conclusions

The lack of awareness of the relationship between clinical patterns and viral etiology could lead to a failure in recognizing COVID-19 cases, and therefore could hamper the ability to provide proper and timely treatment. Given that vaccines are not yet approved for use in children of this age group in many countries, they are currently more vulnerable to infection and the spread of the virus. This study provides valuable insights regarding COVID-19 infection in ambulatory young children. Most cases of SARS-CoV-2 infection in children under two years of age do not require hospitalization, and the majority present only mild symptoms. The principal source of exposure is household contacts, regardless of the implementation of social distancing strategies and lockdown measures. Considering this, our data suggest that day care centers should stay open with adequate measures. Although the use of masks is not recommended for children under 2 years old, other strategies can be used, according to national guidelines, such as social distancing, reducing the number of children in each class, frequent hand washing, and the use of masks by teachers and other personnel, as well as the monitoring of symptoms and case traceability.

According to the variants that circulated during the period of our study, SARS-CoV-2 infection should be suspected in children under two years old who present with fever, irritability, fatigue, and rhinorrhea. Children with a positive household contact and fever should also be tested for COVID-19. The characteristics of SARS-CoV-2 infection in children under 2 years of age found in this study should be interpreted with caution as they only apply to the population included in the study that was done without a control group. Further studies considering contemporary SARS-CoV-2 variants and prevention strategies are needed in order to better comprehend COVID-19 symptoms in very young children. The emergence of co-infection of SARS-CoV-2 with other respiratory viruses, its prognosis, and its affectation on children in their evolution must be kept under observation.

## Data Availability

The raw data supporting the conclusions of this article will be made available by the authors, without undue reservation.
