# Supplementary material for: Clinical and epidemiological characteristics of SARS-CoV-2 virus in ambulatory children under 2 years old
Source: Front Pediatr. 2022 Nov 29;10:957273. doi: 10.3389/fped.2022.957273 (PMC9745022; doi:10.3389/fped.2022.957273)
Supplement: Supplementary file 1 [file Datasheet1.docx]

**Supplementary Material**

**Annex 1 |** Questionnaire for caregivers of children under 2 years of age with COVID-19.

| **Questionnaire for caregivers of children under 2 years of age with COVID-19** | |
| --- | --- |
| **General** |  |
| - Age (months completed) |  |
| - Sex |  |
| - City |  |
| - County |  |
| **Characterization of the clinical features** |  |
| - First symptom at the initial presentation |  |
| - Symptoms during illness | Yes – No – Don’t know |
| - Fever |  |
| - Irritability |  |
| - Fatigue |  |
| - Rhinorrhea |  |
| - Cough |  |
| - Loss of appetite or Difficulty feeding |  |
| - Diarrhea |  |
| - Muscle pain |  |
| - Headache |  |
| - Dysphonia |  |
| - Sore throat or Dysphagia |  |
| - Rash |  |
| - Vomiting |  |
| - Abdominal pain |  |
| - Conjunctival congestion |  |
| - Eye discharge |  |
| - Photophobia |  |
| - Dyspnea |  |
| - Chills |  |
| - Asymptomatic |  |
| - If you had a fever, greater than 39°C? |  |
| **Background** |  |
| - Does your child have any of these diseases? | Premature  Atopic dermatitis  Asthma  Allergic rhinitis  Overweight  Obesity  None of the above  Other: |
| - Has your child used any of these medicines? | Inhaled corticosteroids  Antihistamines  None of the above |
| - Has your child been hospitalized before? | Yes  No |
| - If in the previous question your answer was Yes, indicate the diagnosis |  |
| - In relation to your child's vaccinations, has he/she the national plan vaccinations (free) up to date? | Yes  No |
| - In relation to your child's vaccinations, has he/she received the influenza vaccine? | Yes, complete  Yes, incomplete  No |
| - Have people living in the house been able to maintain preventive quarantine (i.e. prior of getting COVID-19)? | Yes  No |
| - If the answer is No, what activities have they done outside home? |  |
| - Is there anyone living in the house who has been diagnosed with COVID-19, in addition to your child? |  |
| - If in the previous question your answer was Yes, check to whom it corresponds | Mother  Father  Sibling  Other: |
| - If in the previous question your answer was Yes, mark the date the person with COVID-19 living in the house was diagnosed |  |
| - Was your child in contact with any COVID-19 (+) people outside your home? |  |
| - If in the previous question your answer was yes, who was the contact and what is the date of onset of symptoms of the contact? |  |
| **Medical consultation** |  |
| - Consultation date |  |
| - Has your child been given other tests besides that for COVID-19? | Yes  No |
| - If in the previous question the answer was yes, mark the one that corresponds. You can mark more than one alternative. | Panel including other respiratory viruses  Blood count  Chest X-ray |
| - What was the result of the test?   - Panel including other respiratory viruses  - Blood count  - Chest X-ray |  |

**Annex 2 |** Clinical features during illness, in 159 children less than 2 years of age with COVID-19, during the two waves included in the study.

| Symptoms during illness | First wave  n (%) | Third wave  n (%) | P value |
| --- | --- | --- | --- |
| Total | 81 (74.3) | 28 (25.7) |  |
| Fever | 60 (74.1) | 25 (89.3) | 0.940 |
| Irritability | 50 (61.7) | 24 (85.7) | 0.019 |
| Fatigue | 47 (58) | 24 (85.7) | 0.008 |
| Rhinorrhea | 54 (66.7) | 23 (82.1) | 0.121 |
| Cough | 41 (50.6) | 20 (71.4) | 0.056 |
| Loss of appetite or Difficulty feeding | 27 (33.3) | 17 (60.7) | 0.011 |
| Diarrhea | 33 (40.7) | 11 (39.3) | 0.892 |
| Myalgia | 27 (33.3) | 6 (21.4) | 0.000 |
| Headache | 15 (18.5) | 7 (25) | 0.000 |
| Dysphonia | 10 (12.3) | 7 (25) | 0.112 |
| Sore throat or Dysphagia | 15 (18.5) | 15 (53.6) | 0.001 |
| Rash | 15 (18.5) | 10 (35.7) | 0.062 |
| Vomiting | 13 (16) | 10 (35.7) | 0.079 |
| Abdominal pain | 12 (14.8) | 3 (10.7) | 0.000 |
| Conjunctival congestion | 10 (12.3) | 3 (10.7) | 0.818 |
| Eye discharge | 10 (12.3) | 2 (7.1) | 0.448 |
| Photophobia | 12 (14.8) | 5 (17.9) | 0.827 |
| Dyspnea | 5 (6.2) | 2 (7.1) | 0.857 |
| Chills | 11 (13.6) | 7 (25) | 0.255 |
